# Body condition score and its correlation with ultrasonographic back fat thickness in transition crossbred cows

**DOI:** 10.14202/vetworld.2015.290-294

**Published:** 2015-03-07

**Authors:** Randhir Singh, S. N. S. Randhawa, C. S. Randhawa

**Affiliations:** 1 Department of Veterinary Medicine, Guru Angad Dev Veterinary and Animal Sciences University, Ludhiana, Punjab, India; 2 Department of Veterinary Medicine, Guru Angad Dev Veterinary and Animal Sciences University, Ludhiana, Punjab, India; 3 Department of Veterinary Medicine, Guru Angad Dev Veterinary and Animal Sciences University, Ludhiana Punjab, India

**Keywords:** body condition score, back fat thickness, cow, transition period

## Abstract

**Aim:**

The aim was to study the effect of the transition to body condition score (BCS) and ultrasonographic back fat thickness (USG BFT) in crossbred cows.

**Materials and Methods:**

A total of 101 multiparous crossbred cows in advanced pregnancy from organized dairy farm were taken up for study. The cows were grouped according to transition stage, i.e. far off dry (FOD), close up dry (CUD) and fresh (F). BCS was estimated by using the five point visual BCS technique with 0.5 increments. The USG BFT was measured by real-time ultrasound using a portable Sonosite instrument.

**Results:**

In cows with BCS 2-2.5, the BFT of F period was significantly lower than FOD period. In cows with BCS 3-3.5, the mean BFT at F period was significantly reduced as compared to FOD and CUD period. The overall correlation coefficient between BCS and BFT for different transition stages was 84%, 79% and 75% for FOD, CUD and F period, respectively.

**Conclusion:**

The USG BFT gives an accurate measure of fat reserves in cows. The cows with BCS of ≥3.5 entering the transition period are more prone to lose body condition and hence require better and robust management during the transition period.

## Introduction

The body condition scoring (BCS) being a subjective technique is used at regular intervals for assessing the condition of livestock. It is particularly helpful in assessing the body fat reserves of farm animals by visual and manual inspection of the thickness of fat cover and prominence of the bone at the tail head and loin region [1-4]. The BCS system being non-invasive, quick and inexpensive is accepted universally to estimate the degree of fatness [5]. BCS is particularly useful as an aid to dry cow and pre-calving management with the main objective that the cows calve down uneventfully and enter the lactation stage safely [6]. It is also strongly related to milk production and the duration of the postpartum anoestrous interval [7-9]. As the dairy cows use body energy reserves in the early lactation to cope up with negative energy balance [10-15] BCS along with a less common method to assess fat reserves in body tissues i.e. measurement of back fat thickness (BFT) by using real-time ultrasound are more promising approaches to ensure an uneventful transition of dairy cows.

As the cow transition from less demanding non-lactating dry stage to highly stressful lactation stage there is obvious energy and mineral deficiencies and the major outcomes of these deficiencies are metabolic disorders, reduction in body condition score and reduced reproduction efficiency. Therefore, it is needed of the hour to have an efficient and easily applicable tool to estimate body tissue reserves in dairy cows [16].

The BCS provides an easy and reliable method to evaluate the nutritional status, efficacy of feeding system and to assess changes in energy reserves [17,18]. Apart from this, now a days it is widely accepted that the BCS status of a dairy cow indicates nutritional quality, milk yield, reproductive performance, animal well-being and overall farm profitability in a dairy herd [19].

Previously various studies on the precision of BCS system including the ultrasonographic (USG) assessment of subcutaneous back fat indicated that BCS values were closely related to the actual measurement of subcutaneous fat [20]. Few studies were done previously on cross bred cows in India relating to BCS and BFT, but comprehensive information regarding their relation to transition period is lacking.

Therefore, the objective of this study was to examine the relationship between BCS and BFT using real-time USG in transition cross bred cows.

## Materials and Methods

### Ethical approval

All the procedures have been carried out in accordance with the guidelines laid down by the Institutional Ethics Committee and in accordance with local laws and regulations.

### Animals

A total of 101 high yielding multiparous (Milk yield ≥ 25.50 litre/day) crossbred cows in advanced pregnancy from organized dairy farm were taken up for study conducted during August 2013 to September 2014 in Punjab. The cows were grouped according to transition stage, i.e.,

Far off-dry (FOD): > 10 days following dry off and not <30 days prior to calving

Close up dry (CUD): Between 21 and 3 days prior to calving

Fresh (F): 3-30 days in milk

Same 101 cows were followed up throughout the study to evaluate the changes in body condition score and back fat thickness.

### Body composition

**Figure-1:**
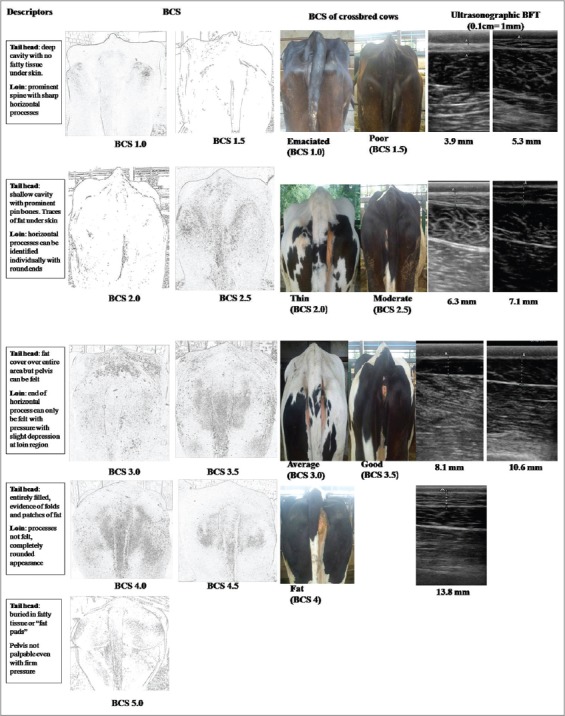
BCS along with principal descriptors and corresponding ultrasonographic BFT in transition crossbred cow

Body condition score (BCS) was estimated by using the five point visual BCS technique with 0.5 increment [21] described in Figure 1.

### Back fat thickness (BFT)

Subcutaneous BFT was measured by real-time ultrasound using a portable Sonosite instrument. BFT was measured in B-mode using 5-10 MHz linear transducer at 7.5 MHz frequency. BFT in the rump or thurl area was measured as the thickness of the layer of sub cutaneous fat between the skin and the fascia trunci profunda located above the gluteus medius muscle. The transducer was placed vertically to an imaginary line between the pins (tuber Ischia) and hooks (tuber coaxe) at the sacral examination site (9-11 cm cranial to the pins) [22] after shaving of site and application of coupling gel (Figure-1).

### Image measurement and interpretation

Images were measured at a depth of 4.7. Captured back fat images were freezed and measured using inbuilt measurement calliper protocol in the instrument.

Both BCS and BFT were estimated on the same day at each stage of transition. BCS and USG BFT was measured at all the three periods, i.e. FOD, CUD and F period in order to observe and calculate any significant change in BCS and BFT.

### Feeding and management

Animals were fed in head to head system in mangers. Feeding involved 45 kg green fodder (Maize, Pearl millet and Sorghum during summer; Egyptian clover and Oats during winter), 6 kg of wheat straw and 2 kg of concentrates per day during last 90 days of gestation. During lactation, the feeding involved 45 kg green fodder, 8 kg wheat straw and 3 kg of concentrates per day.

### Statistical analysis

The USG BFT was presented as mean ± standard error. The statistical analysis was carried out using SPSS (16.0). ANOVA followed by Duncan’s multiple range test was used to estimate significant difference between BFT at different transition period (FOD, CUD and F) at p≤0.05. The correlation between BCS and BFT was estimated by Microsoft Excel.

## Results

The mean USG BFT of cows with different body condition scores for different time periods (FOD, CUD, F) is presented in Table-1. In cows with BCS 2-2.5, the BFT of F period was significantly lower than FOD period but did not differ significantly from FOD to CUD period.

**Table-1:** BFT at different transition stages in crossbred cows for different BCS (mean±SE)

BCS (n)	BFT (in cm)		

	FOD	CUD	F
1-1.5 (05)	0.500±0.14^a^	0.460±0.15^a^	0.418±0.15^a^
2-2.5 (38)	0.591±0.02^a^	0.549±0.02^ab^	0.493±0.02^b^
3-3.5 (54)	0.883±0.01^a^	0.842±0.01^a^	0.782±0.01^b^
4-4.5 (4)	1.12±0.16^a^	1.02±0.14^a^	0.942±0.13^a^
5 (00)	-	-	-

*The values with different superscripts in a row differ significantly at p≤0.05, FOD=Far off dry, CUD=Close up dry, F=Fresh, BFT=Back fat thickness, BCS=Body condition scores, SE=Standard error

In cows with BCS 3-3.5, the mean BFT at F period was significantly reduced as compared to FOD and CUD period. In cows with BCS group 1-1.5 and 4-4.5, the mean BFT reduced from FOD to CUD to F period but did not differ significantly. There was no cow having BCS 4.5 and 5.

Out of 38 cows with BCS 2-2.5, 1 cow at CUD and 5 cows at F periods had a BCS of <2 (Table-2). Fifty-four cows had BCS 3-3.5 at FOD period (Table-2). Out of these 54 cows, 11 and 24 cows reduced to BCS 2-2.5 at CUD and F period respectively. All the four cows with BCS 4-4.5 at FOD period reduced to BCS 3-3.5 at CUD and F period.

**Table-2:** Effect of transition period on BCS in crossbred cows

BCS	FOD					CUD					F				
		
	<2	2-2.5	3-3.5	4-4.5	5	<2	2-2.5	3-3.5	4-4.5	5	<2	2-2.5	3-3.5	4-4.5	5
Number of cows	5	-	-	-	-	5	-	-	-	-	5	-	-	-	-
	-	38	-	-	-	**1**	37	-	-	-	**5**	33	-	-	-
	-	-	54	-	-	-	**11**	43	-	-	-	**24**	**30**	-	-
	-	-	-	4	-	-	-	**4**	-	-	-	-	**4**	-	-

*The bold numerical represent the number of cows with reduced BCS from FOD to CUD and or F, FOD=Far off dry, CUD=Close up dry, F=Fresh, BFT=Back fat thickness, BCS=Body condition scores

The overall correlation coefficient between BCS and BFT for different transition stages was 84%, 79% and 75% for FOD, CUD and F period, respectively.

## Discussion

To the author’s knowledge, this is the first study to evaluate the effect of transition period on BCS and BFT concurrently in crossbred cows. Although studies relating BCS and BFT were done previously, but there was the absence of literature regarding evaluation of the changes in BCS or USG BFT at different transition stages. In the present study the BCS was evaluated at three predefined transition stages in crossbred cows on 1-5 scale with 0.5 increments, as a single BCS does not give any indication of whether a cow is gaining or losing body reserves over a period. Furthermore, BFT was concurrently used in this study to validate the BCS, as during the transition it is difficult to judge accurately the real condition of the animal due to weight gain associated with fetal growth. In our study, the cows with BCS >3.5 were more affected with further change in BCS and BFT in subsequent stages. Similar to our findings Bernabucii *et al*. [23] reported higher reduction in high BCS cows from late pregnancy to first 30 days in milk, than the cows with average and good BCS. This may be attributed to increased resistance of adipose tissue to insulin that predisposes the dairy animal to mobilize non-esterified fatty acid (NEFA), thus potentially creating a vicious cycle of NEFA mobilization and dry matter intake (DMI) reduction during late prepartum period. This is why high BCS animal have lower DMI and more rapid decrease in BCS during the prepartum period than animal of average or good BCS [24].

USG for BFT was evaluated at the most accepted site, i.e. between pins and hooks. Previously Domecq *et al*. [25] tried to quantify fat reserves in dairy cows by examining lumber, thurl, and tail head areas and found highest correlation (r=0.86) between right and left sides of the thurl. Our results show there is strong correlation between BCS and BFT for all the three different stages of transition i.e. FOD, CUD and F. This signifies that USG BFT gives an accurate insight about the fat reserves over a period. Similar findings were reported by Anitha *et al*. [20] with a correlation coefficient of 87% between BCS and BFT in Murrah buffaloes.

In our study we observed that 67.24% (39/58) cows having BCS above 3 reduced significantly to a lower BCS over the passage of time during transition, where as only 15.78% (6/38) cows having BCS 2-2.5 reduced to a lower BCS. Chebel [26] reported that only 24.7% of cows entering the dry period with BCS under 3.5 lost BCS during the dry period, whereas 76.6% of cows over 3.75 lost BCS during the dry period. This difference may be due to the feeding management and environmental differences.

## Conclusion

From this study, it is concluded that cows having BCS 3-3.5 during start of the dry period should be fed balanced energy ration so that they can maintain their condition and cross transition period uneventfully. USG BFT should be concurrently used as an aid to BCS for assessment of body fat reserves in transition cows.

## Authors’ Contributions

SNSR designed the experiment. RS carried out the study along with CSR. SNSR and RS analysed the data and prepared the manuscript. CSR reviewed the manuscript. All authors participated in scientific discussion. All authors read and approved the final manuscript.
